# ST Elevation Sonification of a 12-Lead ECG for the Assessment, Diagnosis, and Monitoring of ST Elevation Myocardial Infarction

**DOI:** 10.3390/s25144373

**Published:** 2025-07-12

**Authors:** Thomas Hermann, Steffen Grautoff, Friederike Tielking, Jan Persson, Hans H. Diebner, Jens Tiesmeier

**Affiliations:** 1 Ambient Intelligence Group, Faculty of Technology, Bielefeld University, 33619 Bielefeld, Germany; 2 Emergency Medical Services, District of Herford, Amtshausstr. 3, 32051 Herford, Germany; steffen.grautoff@klinikum-herford.de; 3 Emergency Department, Herford Hospital, Ruhr-University Bochum, Campus OWL, Schwarzenmoorstr. 70, 32049 Herford, Germany; 4 Department of Anesthesiology, Intensive Care Medicine, Emergency Medicine and Pain Medicine, MKK-Johannes Wesling Hospital Minden, Ruhr-University Bochum, Campus OWL, Hans-Nolte-Str. 1, 32429 Minden, Germany; friederike.brandt@muehlenkreiskliniken.de (F.T.); jan.persson@ruhr-uni-bochum.de (J.P.); 5 Department of Medical Informatics, Biometry, and Epidemiology, Ruhr-University Bochum, 44780 Bochum, Germany; hans.diebner@rub.de; 6 Institute for Anesthesiology, Intensive Care- and Emergency Medicine, MKK-Hospital Luebbecke, Ruhr-University Bochum, Campus OWL, Virchowstr. 65, 32312 Luebbecke, Germany; jens.tiesmeier@muehlenkreiskliniken.de

**Keywords:** auditory display, sonification, process monitoring, parameter mapping, electrocardiography, STEMI

## Abstract

**Highlights:**

**What are the main findings?**

- In a rigorous selection process, an optimal mode for the sonification of ST elevations in an ECG (STEMI) in terms of detection and cognitive processing was determined.
- A classification study with volunteers showed perfect discriminatory power in differentiating pathology-associated ECG signals and high differential diagnostic accuracy in recognizing the severity of STEMI.

**What is the implication of the main finding?**

- The sonification of ST elevations is a significant addition to the diagnostic process, especially in emergency situations.
- The excellent result of the classification is a proof of concept that justifies a randomized controlled trial.

**Abstract:**

We introduce a novel technique for the sonification/auditory representation of a 12-lead electrocardiogram (ECG), the standard diagnostic method for the detection of ST elevation myocardial infarction (STEMI). Our approach to ST elevation sonification conveys the detailed variation of the ST segment to enable differentiated, correct interpretation and severity without consulting a visual display. We present a variety of novel sonification designs and discuss their benefits and limitations. As part of an emergency training program, a cohort of 44 medical students (5th academic year) participated in a classification study in which the diagnostic accuracy of the participants was determined with regard to audibly presented ECG sequences of different STEMI severity levels. Regarding the classification of sonified ECG sequences, the discrimination of isoelectricity (IE, the healthy class) from all other (STEMI) classes combined yielded a perfect classification of all 660 classification instances (sensitivity = specificity = 1). With respect to the individual classification of all five classes (IE, inferior/anterior, and moderate/severe STEMI), an overall accuracy of 0.82 (0.79, 0.85) and an intraclass coefficient of κ=0.77 were estimated.

## 1. Introduction

Acute chest pain accompanied by suspicion of ST elevation myocardial infarction (STEMI) is common in Europe. The incidence of hospital admissions for STEMI is between 44 and 142 per 100,000 inhabitants per year [1]. Emergency Medical Services (EMSs) are involved in most cases for diagnosis, initial treatment and transportation [2]. The 12-lead electrocardiogram (ECG) is the diagnostic cornerstone of STEMI [3].

According to a recent study, sonification—the scientific method of representing data as sound in an auditory display—has the potential to play an important role as a new supporting tool for ST-segment surveillance during out-of-hospital care for patients suspected of having myocardial infarction [4].

**Figure 1:**
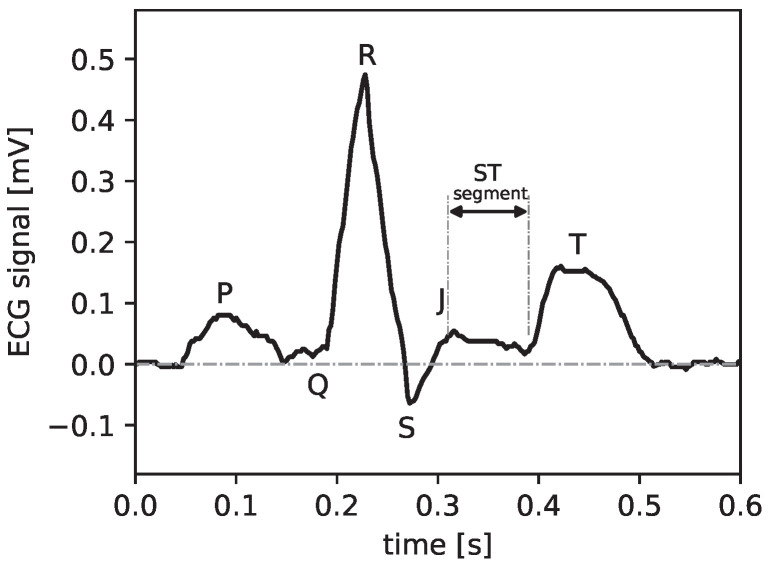
ECG signals with moderate ST elevation; data is typical for what was used for the sonification presented later.

Sonification converts data, particularly biosignals, into sounds in a systematic and reproducible way. In our context, the ECG is used as a data source [5]. These sounds are designed to convey information about biosignals to the EMS crew. Other examples of the auditory display of biosignals include pulse oximetry and the QRS tone of an ECG (cf. Figure 1 for the notation of ECG signal segments), which are established methods in the medical environment. In the case of an ECG, the ST segment (Figure 1) can also reveal different sounds when it is isoelectric or elevated [4].

Rhythm disturbances are monitored continuously by one to three leads to enable the immediate treatment of malignant cardiac arrhythmias when they occur. According to the European Society of Cardiology (ESC) clinical practice guidelines, a 12-lead ECG has to be recorded on-scene within ten minutes of arrival but routinely recorded only once. The evaluation results of the ST segments are dependent on the age and sex of the patients, particularly the leads V2 and V3, according to the current ESC guidelines [3]. Therefore, good knowledge and training are essential to ensuring correct and prompt diagnoses.

The diagnosis of STEMI can be challenging for EMS in cases of fluctuating symptoms due to transient ECG changes [6]. We explore the potential of sonification to support EMSs in making the correct diagnosis by distinguishing ST elevation from non-ST elevation during the prehospital surveillance period. A quick and correct classification is necessary because if ST elevation or other signs of coronary occlusion are present in the ECG, immediate revascularization is required. Treatment includes the prompt delivery of medication on-scene and urgent heart catheterization. Therefore, the treating hospital has to offer 24/7 acute revascularization therapy [3].

Encouraged by the recent findings of a feasibility study [4], we formulated the following hypothesis for the current study: The sonification of the ECG can provide interpretable information about ST segment elevation and can be used supportively for emergency medical use. To test this hypothesis, we first developed a sonification concept to create an auditory representation of different STEMI severity levels and types. This concept incorporated evidence taken from corresponding cognitive studies to optimize information gain by reducing unnecessary cognitive load as much as possible, and we subsequently run through an expert evaluation process. We then conducted a classification study on a descriptive level with a training cohort of students. Of note, the presented classification, showing high accuracy, is only the first module of a bi-modular study. This first part is regarded as a preparatory step toward a confirmatory study addressed in the second part, a randomized controlled trial [7]. A real-world study is our overriding near-term goal. We have prepared such a real-world study within the framework of ethical feasibility.

## 2. Related Research

A number of methods have been developed for the sonification of uni- and multivariate biomedical time series, such as the sonification of electroencephalography (EEG), of electromyography (EMG) and of CT/PET scans, to name a few [8,9,10,11]. With respect to ECG sonification, Kanev et al. have summarized the state-of-the-art in ECG sonification [12]. There is recent work on novel wearable devices integrating ECG and PCG for cardiac health monitoring [13,14]. While data is displayed mostly visually so far, we regard wearables and mobile devices as highly promising for sonification applications. Current work on ECG sonification splits into two research lines. The first group of approaches focuses on sonifying temporal features such as the heart rate and the heart rate variability [15,16,17]. For instance, heart rate sonification supports better self-regulation in sports training intensity [18]. In contrast, heart rate variability is an early diagnostic indicator of arrhythmia and other heart-related diseases [17].

The second line of research focuses on the representation of the morphology of ECG signals, supported by the fact that pathological states usually correspond to specific changes in the ECG signal shape—with applications to facilitate and support diagnostic tasks [19,20].

In summary, ECG sonification is a recognized field, especially when it comes to monitoring and detecting cardiac pathologies. To our knowledge, however, more elaborate/detailed sonifications are yet in the state of basic research, as complex sonifications are usually not integrated in medical devices or professional procedures.

Previous research introduced ECG sonification, with a focus on the ST segment. Its elevation was represented by various sonic means, such as using a formant synthesizer for *polarity sonification*, to synthesis water drop sounds for the so-called *water ambience sonification*, or a subtle morphing between timbres in morph sonification [4,5]. These approaches provided an overall indication of whether the ST segment was elevated but did not enable perception of the spatial distribution, i.e., in which orientations/projections ST elevations or depressions occur. To make these spatial patterns stand out clearly, the present study introduces an approach similar to what is known as *temporal nesting* in the field of parameter-mapping sonification [21].

Another focus of our previous ECG sonification research was the development of an *auditory magnification loupe*, which perceptually magnifies changes in a variable, making subtle value changes highly salient. For instance, subtle rhythmic irregularities that are too small to be perceived as a change in rhythm have been mapped onto pitch deviations, thereby making them highly salient cues [16]. Although we are taking a different approach here, this method could be a promising option for future projects. For example, it could be used to emphasize relative changes compared to a reference ST elevation profile.

## 3. Conceptual Design of ST Elevation Sonification

In this section, we present the key ideas for the ST elevation sonification. This includes relevant data properties, such as feature extraction, as well as sonification techniques, including novel designs that anchor the sonification to the QRS tone, which serves as a baseline auditory display in emergency settings. We here focus on the conceptual level and address aspects of software architecture, sound computing and the embedding of the sonifications into the acoustic ecology in Section 4 and Section 5.

### 3.1. Electrocardiography

The ECG is the (usually visual) display of potential differences in cardiac muscle cells over repeated cardiac cycles. The potential differences are measured via electrodes placed on the skin. There are standards for depicting the resulting set of curves as amplitudes over time. The voltages depend on various factors such as the thickness and activation of the heart muscle, the distance between the electrode location and the heart, and the type of the surrounding tissue (e.g., fluid or fat) [11]. Usually, more than two electrodes are used, and thus the electrical activity can be assessed from different angles (i.e., projections), which provide clinicians with relevant information for (mal-)function [16]. The standard ECG uses 12 leads, including 3 limb leads, 3 augmented limb leads, and 6 precordial leads, thus offering rich spatial information.

Concerning the temporal structure of the ECG, the time series can be divided into the following parts, which represent different states of the heart’s cycle (see Figure 1). The periodic repetition of depolarization and repolarization causes a contraction, followed by relaxation of the heart muscle. (i) The P wave results from the depolarization of the left and right atrium; (ii) the QRS complex represents the depolarization of the ventricles, whereby the repolarization of the atria is usually masked by the QRS complex; and finally, (iii) the repolarization of the ventricles manifests as the T wave.

References (P, Q, R, J, S, T) are usually used to pinpoint heart abnormalities. In particular, the ST segment, i.e., the J point, the beginning of the ST segment, may deviate from the ‘healthy’ state, which is ‘isoelectric’ (IE), i.e., exhibits neither elevation nor suppression. Deviations from that isoelectric state could be caused by a blockage in the coronary arteries. These conditions are important for the subsequent feature selection and proper choices for the sonification method to maximize diagnostic value for assessing STEMI conditions.

### 3.2. Feature Extraction

**Figure 2:**
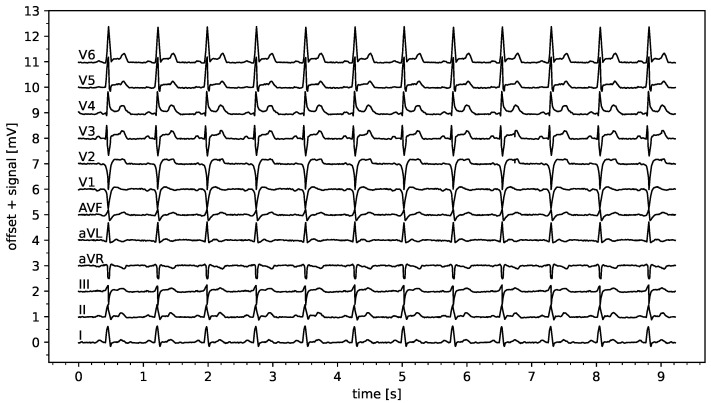
ECG data set depicting a 10 s 12-lead recording of a mild anterior STEMI.

We use a 12-lead ECG as our data resource, which we extract from simulation runs of the ECG generator of the Laerdal Resusci Anne Advanced SkillTrainer training device. We chose this data source because we will conduct a subsequent user study to evaluate a selected sonification in a scenario study, for which a patient mannequin will be used and Laerdal will be used to compute and present real-time data (to be published elsewhere). For brevity, we omit technical details on extracting the raw data, as they are irrelevant here. We assume 12 signals (leads) to be given as time series $x_{k}\left(t\right)$ for lead k=1,…,12. Furthermore, we assume that all signals have been appropriately band-pass filtered, with values of 0 corresponding to isoelectric states and no high-frequency noise polluting the readings. Figure 2 depicts one of the datasets that we use for feature extraction.

**Figure 3:**
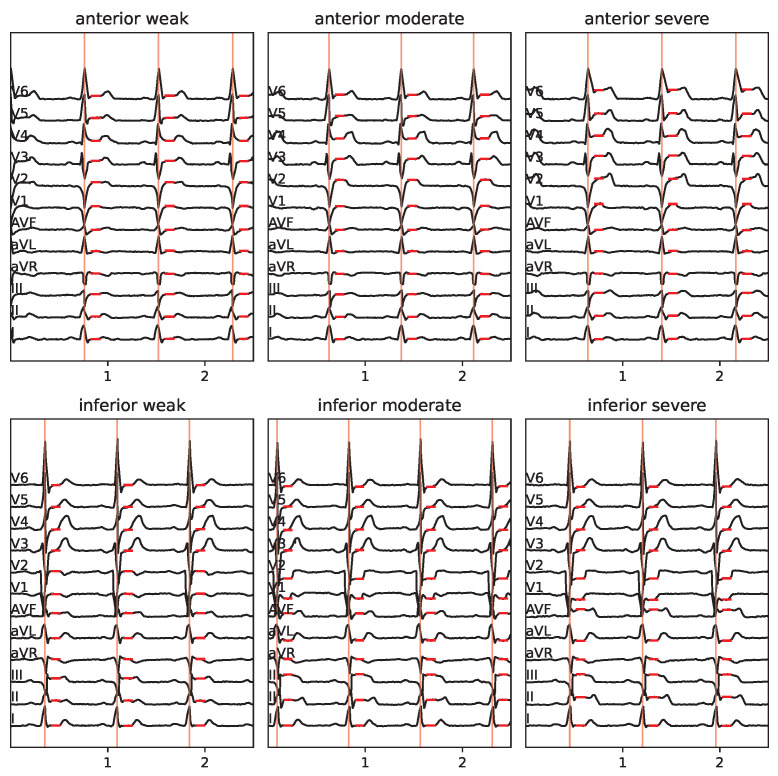
ECG signals extracted from Laerdal simulations for the cases indicated in the panel headers (x-axis shows time [s], y-axis the signal in arb. units). Vertical red lines depict our R-peak detection results, and horizontal red line segments depict our ST elevation estimations, as explained in Section 3.2.

As our main objective is to develop a method of conveying information about STEMI, we used the Laerdal ECG generator to create ECGs according to two controlled parameters: direction (anterior vs. inferior) and severity (weak, moderate, severe). This resulted in 2×3=6 data sets in total, seven when including the IE condition. To keep the study focused, we did not vary the heart rate, adjusting it to 80 bpm for all datasets. Excerpts from the two-second duration of the resulting six datasets, each duration of two seconds is depicted in Figure 3.

Assuming properly calibrated readings in terms of μV/unit, the key features here are the ST elevations. These features are extracted from the raw data using the following method: First, the locations of the R-peaks are identified. To this end, $s\left[n\right]=\sum_{k=1}^{12}s_{k}^{2}$ is calculated and rescaled to the range r=[0,1]. Second, we utilize the peak finder algorithm peak_find() from Python/SciPy, setting the peak height to 0.5 and the peak distance to 30% of the sampling rate. This is then resampled (down) to 100 Hz from the given data at 500 Hz. Peaks are accurately discovered in the given data; however, we acknowledge that a more elaborate approach might be required for arbitrary real-time data from real sources, which is beyond the scope of this study. Figure 3 depicts excerpts of the extracted R-peak locations.

For the datasets at hand, we extracted the ST elevation (or suppression in the case of negative elevation) by calculating the median value for each channel within the time interval of the heartbeat. This means that 0% and 100% refer to the current and subsequent R-peak time points, respectively. The resulting ST elevations are depicted as red horizontal line segments in Figure 3 and align with evaluations of accurate ST estimates by medical experts (the selected authors of this article). For pragmatic reasons, we refrain from discussing a more generally applicable feature extraction method in order to maintain the focus on feasible sonification designs.

**Figure 4:**
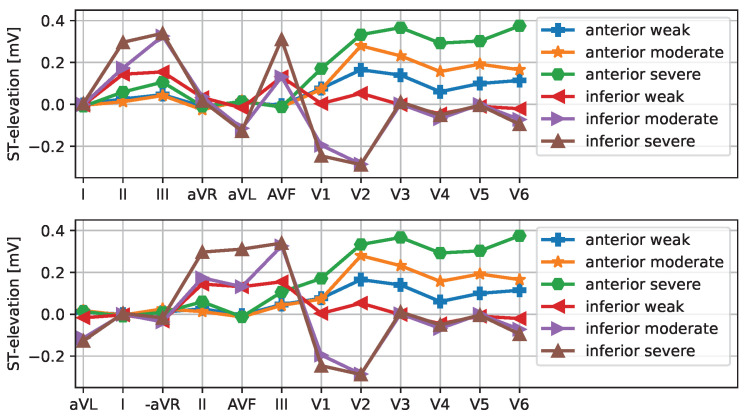
ST elevation vectors for the six selected cases are shown as a parallel coordinate plot. The upper plot shows the limb leads in standard order. The bottom plot shows the channels along the Cabrera circle with the aVR lead inverted/mirrored.

While the depiction of ST displacements in Figure 3 appeals to medically trained users, we also present the data as parallel coordinate plots (Figure 4) to make ST elevation profiles clearer. Note that the order of the leads (limb leads followed by precordial leads) does not correspond to a neighborhood relation; rather, the connection of the points by solid lines is used to facilitate perception. Where appropriate, the limb leads are ordered as follows: aVR, III, aVF, II, I, aVL (i.e., from inferior to lateral) or alternatively aVL, I, −aVR, II, aVF, III.

The sonification designs introduced in the following section are based on these 12-dimensional ST elevation vectors.

### 3.3. Sonification Designs

The purpose of the sonifications is to provide detailed information about the ECG within STEMI situations, particularly in emergency settings where physicians must juggle many competing tasks, people, and displays. Therefore, the designs should produce sounds that are as salient as necessary, yet as unobtrusive as possible. Verbal cues (a type of auditory display) are not applicable in this context due to the disruption they would cause to verbal communication between team members, between the hospital and the team, and between the team and the patient. Auditory information is superior to visual information because sound is ambient, unlike visual displays, which require crew members to look at them. As the ECG is already the point of reference and iconically connected to the beep sound of the QRS tone, it is a natural and proven choice to anchor the auditory representation to this sound stream and augment it with detailed information that is intuitively associated with the heart’s electro-mechanical condition.

There are multiple sonification techniques, including *audification*, *model-based sonification*, *wave space sonification*, *parameter-mapping sonification*, *parameterized auditory icons* and *earcons*, to name the basic categories [22]. Each method has specific applicability conditions, such as the amount and structure of the data. Given the 12-dimensional vectors, parameter mapping sonification (PMSon) (cf. [21]) is the most appropriate choice. PMSon is a highly flexible method of connecting analog data, such as the continuous ST elevation values, with sound synthesis parameters that shape the perceptual qualities of sound events, such as pitch, sharpness and loudness. Choosing sound events that correspond to meaningful sounds in the world allows one to connect PMS with auditory icons, hence creating hybrid approaches. Below, we introduce and present a number of designs focusing on *analogic representations* [23]. This means that the variation of ST elevation should cause perceivable variations of the corresponding sound. This contrasts with *symbolic representations* that condense the raw data into one or a few semantic units conveyed as notifications that require learning. The motivation behind analogic sonification designs is to draw the users’ attention to significant changes as soon as real-time data allows in order to recognize incipient transitions towards critical situations and avoid their manifestation. It is noteworthy that automatic classification is biased on false positive results and may therefore mislead users. Furthermore, it has previously been demonstrated that analogic auditory representations used to assist with secondary tasks within the scope of process monitoring can switch the operational mode from problem solving to problem anticipation [24].

#### 3.3.1. Sonification Concept 1: Heartbeat-Locked ST Elevation Arpeggio

In practical ECG recordings, a new 12-dimensional ST elevation vector will be available for each heartbeat. One way to inform users with the least possible latency is to append sonification directly to each QRS tone. For subsequent demonstrations, we will play a short noise burst to mark the onset of the QRS complex. This will enable to use the display alone or later as an addition to existing heart monitors. We propose generating a sequence of 12 tones, played in rapid succession, merging into an arpeggio. Each tone will represent one lead, appearing in the order as plotted in Figure 4, with a musical pitch along a musical scale to be chosen. For example, one option is a sequence of 12 tones along a pentatonic scale. Another option is to use six tones along a diatonic scale for the limb leads, with the same six tones transposed up by one octave for the precordial leads. As an alternative, the 12 semitones of an octave could be used; however, this would not produce the desired aesthetic sound. Obviously, this design concept offers multiple modes for mapping the available data to sonic variations, thus transforming data into musical structures. A few sub-designs deserve special mention:

Design S1.1: tones are played longer and louder if the corresponding lead is elevated or suppressed, dull (respectively, medium or sharp) for a suppressed (respectively, isoelectric or elevated) ST segment:

- With a diatonic sequence [0, 2, 4, 5, 7, 9, 12, 14, 16, 17, 19, 21], i.e., major scale, precordial leads transposed by one octave (i.e., 12 semitones offset): Sound examples (sound files are provided as Supplementary Materials) S1.1a.wav for the six STEMI conditions [weak anterior, moderate anterior, severe anterior, weak inferior, moderate inferior, severe inferior], three heartbeats for each condition.
- With a pentatonic scale [0, 2, 4, 7, 9, 12, 14, 16, 19, 21, 24, 26]: Sound examples S1.1b.wav for the six STEMI conditions as in the previous item.

The design patterns are clearly discernible, since an isoelectric ST in all channels results in a rising arpeggio of short unobtrusive tones, whereas individual non-isoelectric notes stand out. Affected leads are identified by pitch and location in sequence. The octave suggested in variant (a) is intended for musically trained listeners and provides another cue for distinguishing between limb and precordial leads.

Design S1.2: percussion sequence: In this design, the channel is solely identified by its position in the sequence. Consequently, the pitch is free to convey the ST value, which is quantized as below −θ, within the range of [−θ,θ], or above θ. Three drum sounds of increasing drum tension are used to represent ST elevations ranging from suppressed to elevated. The neutral sound (ST around IE) was given a much higher damping so that it would not dominate the sound. We found that inter-tone intervals of dt=40ms, as used with the previous method, are too to discern the rhythm. However, with higher inter-beat intervals that better suited our ability to perceive rhythms in music, the 12-beat sequence would often extend beyond the onset of the subsequent heartbeat. Yet even with very fast sequences, it seems to be possible to discern roughly where the accents are located (i.e., limb or precordial leads), how many there are, and whether they are elevated or suppressed, in the corresponding parts of the vector. The sound example S1.2.wav illustrates the percussion sequence for the six conditions, showing three heartbeats with a 1.5 s gap between each condition. Its repetitive nature likely reduces its suitability for use in permanently playing process monitoring over extended periods of time.

Design S1.3: Vowel Transitions: In this design, we adapt the design S1.2 by replacing a sequence of 12 drum hits with a single vocal utterance. ST values (again quantized into three levels—suppressed (below −θ), around IE, and elevated (above +θ)) are mapped to German vowels: ‘o’ (as in bow), ‘a’ (as in bath), and ‘i’ (as in bee). We used the *Vowel* class in Supercollider [25] to compute formants for a male bass voice excited at $f_{0}=80$ Hz, smoothing transitions (implemented via blend() by means of the SuperCollider Lag Ugen). The sound example S1.3.wav presents the auditory representations of three heartbeats for each of the aforementioned six conditions, separated by a 1.5 s gap. The continuity of the sound streams reduces the required level of attention. Being in close proximity to verbal sounds activates one’s own language processing skills and may aid in the memorization of patterns compared to rhythms or melodies. However, articulatory speech sounds can interfere with vocal interactions between EMS personnel and affect mobile phone conversations, as they are probably not properly filtered out by environmental sound filters.

In summary, all of the aforementioned designs are rich in information and capable of conveying beat-to-beat variations of the ST profile. However, this results in the generation of highly repetitive sound chunks that may become quite boring or intrusive with extended listening. Also, assuming a resting heart rate of 80 bpm, the speed of the arpeggios/rhythm is already high. This may not be ideal for sustained use. Therefore, we propose the following alternative paradigm.

#### 3.3.2. Sonification Concept 2: Musical Phrase Melody over Several Heartbeats

**Figure 5:**
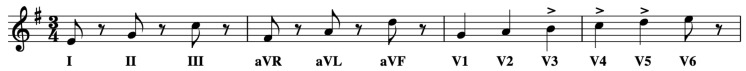
Musical representation of ‘a sonification melody’ to disperse information over several heartbeats (QRS tones played at the bars).

While developing Concept 1, it became clear that the heartbeat could serve as a bar in the same way that a metronome would in musical notation. We propose composing a musical motif that spans several bars to convey a ST profile over a longer/extended time interval. This means that the ST vector will only be updated every *k* heartbeats, where *k* is a small number (e.g., k=8). Specifically, the sheet music in Figure 5 illustrates a typical melody with a 3/4 (waltz) rhythm, similar to that of a minuet.

Note that aspects such as note length (e.g., 1/4 vs. 1/8) or accents can be assigned according to the actual ST elevation present in the relevant channel. Notes can even be replaced by silence (i.e., a pause) if the lead’s ST segment is isoelectric. As a constantly repeating melody can quickly become boring or annoying, one could reduce its frequency, e.g., playing it every 20th heartbeat or once per minute. Alternatively, one could play it at a higher frequency if there is a clear deviation from a reference, for example, the initial visual inspection of the 12-lead ECG.

Using musical instruments such as the cello enables different phrasing techniques to be employed, such as playing a note quietly and percussively for low absolute ST (pizzicato) and playing the note bowed and loudly for high absolute ST (arco/marcato). The Sound Examples S2-*.wav illustrate this approach for the six selected conditions of STEMI: weak anterior, moderate anterior, severe anterior, weak inferior, moderate inferior, and severe inferior. Note that, in the event of ST suppression, the cello notes for leads where abs(ST) exceeds 0.1 mV are transposed one octave down.

There are many possible variations, such as using different musical instruments for leads 1–3/aVR aVL aVF/V1-V6, or for elevated or suppressed leads. For example, you could use a piano for the limb leads, a wind instrument such as a flute for the augmented leads and a string instrument such as a cello for the precordial leads. Such a multitimbral approach might help listeners attribute sounds more easily, but it requires them to learn the associations. The mappings as proposed here fall within the field of parameterized earcons: motifs and timbre selection are key elements; however, the mapping of data features to phrases lies between *analogic* and *symbolic* representation, essentially enabling the sound to ‘speak for the data’.

On the one hand, this procedure could be acceptable for practical use; on the other hand, it might be considered too playful and not serious enough, as it relies on the memory of a specific melody. In our experience, we dislike melodies that get stuck in our heads, such as the wake-up melody or ringtones designed as audio branding for mobile phone companies. For this reason, we have decided not to pursue this approach but rather to focus on the third and last design for this paper.

#### 3.3.3. Sonification Concept 3: Grouped Lead Scans

**Figure 6:**
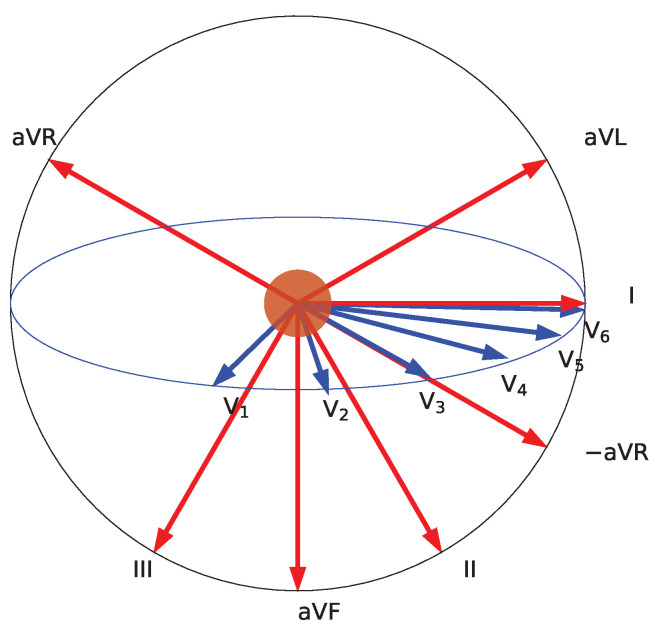
Lead projections along the Cabrera circle (red) using mirrored aVR as −aVR. The red circle represents the heart, the patient’s body is upright and facing towards the reader. The ultimately selected sonification progresses from aVL to III (red) in the first group, followed by V1 to V6 (blue) in the second group. Lead aVR is also shown, as it is used in some sonification designs.

The key idea for the next stage is to create a sonification that sits between the first design, which includes all the data for each heartbeat, and the second, which is a highly distributed representation. This involves using sufficient time, but no more than is necessary, for the targeted representation. Additionally, we aim to keep the sound as simple as possible to avoid overstraining the memory of the melody. In our assessment, this level of detail is compatible with a typical duration of about 10–15 s, during which time the melodies can be memorized. Therefore, the best choice for designing the display in terms of cognitive load is to leave time intervals between repetitions silent. As a refinement to the limb/augmented/precordial order, we decided to use the Cabrera circle for the limb and augmented leads, i.e., the order aVR, III, aVF, II, I and aVL as depicted in Figure 6. This gives two groups of six elements each, which also fits with the number of items that can be easily remembered (five to seven).

We align the presentation of these groups with the QRS tone of a heartbeat, using this as a trigger. However, we keep the inter-tone interval constant and independent from the heart rate, setting it at the most ideal rate possible for scanning. We acknowledge that listeners may initially prefer a low scanning rate but may then wish to increase it as they become more familiar with the sonification. This is similar to how visually impaired users speed up their screen readers and how language learners become able to decipher faster speech as their skills improve. Because the position in the sequence already determines the lead, we reserve pitch—arguably the most salient perceptual quality of a tone—for displaying ST elevation. This approach is supported by the fact that most listeners intuitively associate pitch with vertical displacement, viewing higher pitches as being higher above the ground. While we acknowledge that this may not be universally applicable, it is probably the dominant association. For this reason, we adopt it and refer to Walker’s studies on polarity in mapping (cf. [26] for further details). However, representing ST elevation by pitch requires the definition of a reference pitch—i.e., a pitch representing isoelectric ST—because pitch is a continuous attribute without an inherent zero point. We can establish a clear reference point by using the pitch of the QRS tone. ST tones higher (respectively, lower) than this should represent ST elevations (respectively, suppressions). This still leaves a large design space with various options, such as whether to use discrete or continuous values for the mapping. These variations will be illustrated below. As to the intensity, we acknowledge that the ST elevations will naturally exhibit some variability. Consequently, there will be a threshold below which elevations or suppressions are possibly clinically irrelevant, such that related variation in the sonifications may rather mislead or irritate the users. In contrast, large elevations and large suppressions should stand clearly out, which can be implemented by using a nonlinear mapping to sharpness and/or modulation and/or sound level. In summary, the sonification scheme reads as follows:

- On each heartbeat play the QRS tone.
- Every *k*th heartbeat (e.g., k=10) plays a sequence of six notes for limb leads in the order aVR, III, aVF, II, I, aVL, via inferior to lateral, with pitch encoding ST level, relative to the pitch of the QRS tone. In addition, outside a ‘healthy’ range, larger sharpness, or modulation, is used.
- On the heartbeat k+d (*d* = 1, 2, or 3 adjustable), play six notes for the precordial leads V1, …, V6, i.e., from septal via anterior to lateral, using the same mappings as for limb leads. Optionally (if necessary), apply a different timbre if users cannot distinguish the groups.

This procedure defines an *absolute mapping*. Alternatively, it could be defined as a *relative mapping* by assigning the pitch value for each lead relative to the lead’s ST elevation recorded initially via the 10 s ECG as the reference.

In order to explore the still large space of possible sonification mappings, we constrained the design space by introducing sonification hyperparameters that could be used to configure sonification. The first of these refers to the pitch mapping used for specific designs. We regard spectral (or pitch) mapping as the most important factor, i.e., how ST evaluation will be mapped to the pitch of a tone. We considered and implemented the following variants:

- Continuous mapping: The ST elevations are mapped linearly to pitch, corresponding to an exponential mapping to frequency. A suitable source interval is [−0.4, 0.4]μV, a suitable target interval is a musical fifth, i.e., seven semitones on the chromatic scale down and up, i.e., [−7, 7]; cf. Section 4 for details on the implementation in Python.
- Chromatic mapping: starting from the continuous mapping described above, here we round (i.e., discretize) the pitch value (represented in MIDI notes as floating) to integer values, resulting in pitches as they occur on the chromatic scale, i.e., tones available on the piano keyboard (though we do not need to use any specific musical instrument).
- Diatonic mapping: Here, we choose all tones of two octaves (excluding the octave below and above the center tone) and map the source interval [−0.4, 0.4]μV to the index 0,…,11 in this array of notes; thus, we obtain a musical interval (when relating the tone to the QRS pitch), which we are highly familiar with from Western music and which are rather easy to recognize: tonic, major second, major third, forth, fifth, major sixth, and major seventh.
- Five-state mapping: Here, we discretize the ST elevation into five levels: severe suppression, weak suppression, IE, weak elevation, and severe elevation) using two thresholds ($\theta_{1}$, $\theta_{2}$) and the sign of the value for group selection. For pitch mapping, we experimented with a harmonic choice, i.e., the tones should fit a major chord, choosing the tones e, g, c’, e’, and g’ from negative to positive, or [−9, −5, 0, 4, 7] as the semitone offset to the pitch of the QRS tone.
- Fivee-state-dissonant mapping: This is a variation of the previous five-state mapping, but here, we chose the extrema of the pitches to be dissonant to elicit a clear feeling of discomfort, i.e., such sounds should draw more attention as they are more disturbing or unpleasant (when heard together with the QRS tone). Specifically, we tried a major seventh down and a minor sixth (augmented fifth) up; in particular, the major seventh down has much friction with the reference.
- Tristate mapping: Here, we discretize the ST elevation in just three niveaus: For each specific ST elevation, *s* we use
  - For s<−θ: a lower pitch, e.g., a forth below the reference;
  - For s∈[−θ,θ]: the reference pitch;
  - For s>θ: a higher pitch, e.g., a fifth above the reference.

The next sonification hyperparameter is the channel mode, which determines how channels are organized. They can be organised either along the Cabrera circle (i.e., in the order aVL, I, −aVR, II, aVF, III) or counterclockwise (i.e., in sequence avR, III, aVF, II, I, aVL).

Three further parameters refer to the temporal organization: (i) Inter-tone interval dt [s] controls how quickly the tones play in both the limb and the precordial group, (ii) *Stride* controls the gap (in heartbeats) between sonification initiations. (iii) *Separation* specifies the gap (in heartbeats) between the limb lead and the precordial lead group. Through experimentation, we identified two feasible settings. Firstly, a slow setting (dt=0.2 s, separation = eight heartbeats, stride = two heartbeats), which leaves enough time for the listener to cognitively process the meaning conveyed by each tone and gap before the cycle begins again. Secondly, a fast setting (dt=0.08 s, separation = four heartbeats, stride = one heartbeat), where all tones are essentially presented between two heartbeats (at least at 80 bpm). While all settings in between are of course possible, the two selected settings correspond to the two modes: novice (learner) mode and expert (pro listener) mode.

Finally, the overall level of the tones relative to the QRS tone level needs to be adjusted in decibels. We attempted this by using a dB offset; however, we added a parameter dBscale to allow the more precise adjustment of the loudness mapping.

The following Supplementary Materials contains sound examples for the proposed pitch mapping modes: continuous, chromatic, diatonic, five-state, five-state dissonant, and tristate. Furthermore, it contains samples corresponding to the five-state mode sonifications using different speeds (where dt specifies the time between notes in seconds), stride (six and eight), and set separation (one or two).

- The 30 sound examples S3.1–S3.30 are titled in a self-explanatory way, specifying mode, condition, dt, stride and separation. We recommend listening to files in blocks of six that belong to the same mode and only vary in condition.
- Sound examples S3.31–S3.34 all correspond to a single selected condition (inferior moderate) but with different dt, stride, and set separation. For instance, sonification example S3.34 is fast and dense and may be the setting of choice if ST elevation changes are expected and should be detected no later than three heartbeats. S3.31–S3.33 show a slightly faster lead sequence (dt=80 ms) with different modes.

We observed that with increasing familiarization, faster settings can be well processed.

## 4. Implementation of ST Elevation Sonification

For the purpose of real-world medical applications, we eventually target the integration into standard ECG recording devices in cooperation with device manufacturers. However, for method development and testing, a standard computer platform offers higher flexibility and facilitates interaction with and plotting of data. We use Python 3.10 with *numpy/scipy/matplotlib* for data processing and plotting and our self-developed *mesonic* Python package as middleware for parameter mapping sonification [27]. With *mesonic*, we abstract from the sound rendering backend, but we utilize *SuperCollider3*, a powerful sound programming system, for sound rendering. As interface between mesonic and SuperCollider3, our self-developed Python package *sc3nb* [28] is used.

**Figure 7:**
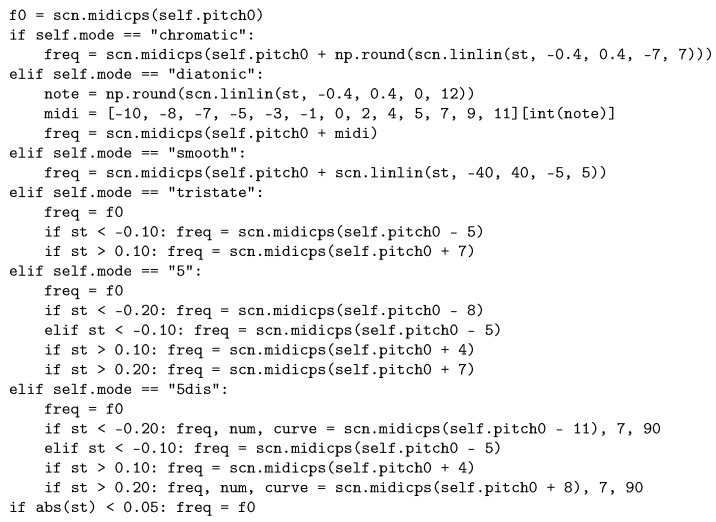
Code snippet for the various pitch mapping modes in ST elevation sonification design 3.

The Supplementary Materials contains a Python program source as a Jupyter notebook, providing the code to render sonifications according to the design used in the study. The program takes as input the ST profile, i.e., the vector of limb/augmented leads along the Cabrera circle and the precordinal leads. The  hyperparameters *set separation*, *stride*, *f0*, and *heart rate* can be adjusted for experimentation. Data for the six STEMI conditions (anterior and inferior, weak, moderate, and severe STEMI) are included in the source file. The code snippet in Figure 7 illustrates how ST elevations are mapped to pitch and subsequently to audio frequencies according under the different pitch mapping modes. Note that midicps converts pitch (as MIDI notes) to fundamental frequencies in Hz and the linlin$\left(x,s_{1},s_{2},d_{1},d_{2}\right)$ function maps the *x* from $\left[s_{1},s_{2}\right]$ to $\left[d_{1},d_{2}\right]$.

## 5. Sonification Demonstration and Method Selection for Evaluation

**Figure 8:**
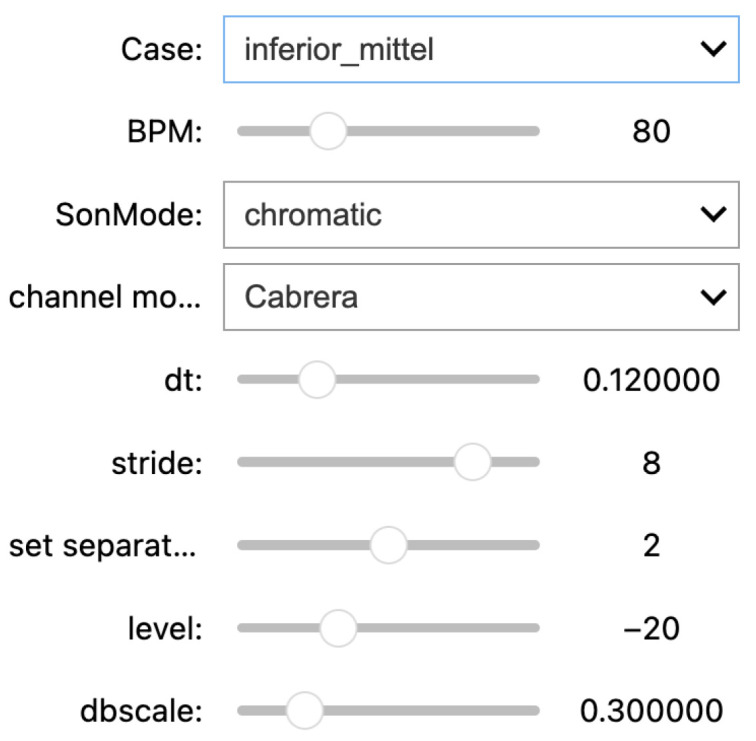
User interface to explore the parameter space of mappings within the sonification designs of concept 3. Parameters are (from top to bottom) (i) STEMI condition (cf. main text), (ii) heart rate (bpm), and hyperparameters to configure the sonification being (iii) the spectral mapping mode (chromatic, diatonic, 5-state, tristate, 5-dissonance), (iv) the channel mode (Cabrera vs. counter-clockwise), (v) the inter-tone interval dt, (vi) the gap between sonification initiations (stride), (vii) the separation of groups of 6 channels in heartbeats (separation), and (viii) the overall level of the tones relative to the QRS tone level in dB and (ix) dBscale (finely calibrating the loudness mapping).

The sonification methods introduced above demonstrate how flexible and versatile sonifications can be crafted, even under the narrow constraint of using just the 12-lead vector of ST elevations and even when narrowing the methodical focus even further to PMSon-based designs. Due to the sheer amount of possible designs, a comprehensive evaluation of all variants is not feasible. Therefore, design criteria and validation are based on expert reviews involving clinical partners, coauthors, and selected colleagues with different expertise. For that purpose, we developed a real-time sonification player and demonstrator that allows the user to interactively select sonification hyperparameters via drop-down widgets and sliders, as depicted in Figure 8.

**Figure 9:**
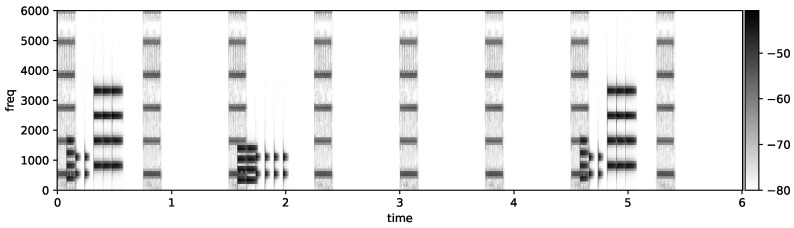
Short -term Fourier Transform of the inferior-severe sonification using 5-state-mapping mode: after QRS tone 1 and 7, we see the 6-limb lead ST elevation tones, and after QRS tone 3, we see the weaker precordial ST elevation tones.

Figure 9 depicts a spectrogram of a 5-state sonification for the ST elevation profile of an severe inferior STEMI, using the parameters dt=80 ms, stride =6, set separation =2.

The key criteria for selecting the design and setting suitable hyperparameters, i.e., for model selection, were as follows.

**Comprehensibility:** The five pitch levels are few enough to be understood correctly without much training, and the low presentation speed allows one to follow the pitch contour.
**Dominant Perceptual Quality:** The presentation speed is fast enough that the six channels in each block are perceptually grouped in one cluster, forming a pattern or gestalt, similar to how raindrop sounds fuse into the perception of rain, or a sequence of knocking sounds merge into the interaction of a woodpecker with a tree. This grouping facilitates the association of groups to lead groups. Users are thus not challenged to interpret individual leads.
**Aesthetics/pleasantness:** The sound should be pleasant to hear, e.g., not too frequent, not too harsh or loud. This motivated the selection of dB, musical intervals for the two elevation pitches, and the two suppression pitches. The isoelectric pitch is already given by the pitch of the QRS tone.
**Memorizability:** Memorizability is an important characteristic optimized with design 3, as it involves only two groups, and the sequence is topologically ordered (Cabrera circle resp. counterclockwise)
**Compatibility with environmental sounds:** Note that the selection of sounds as synthesized sounds instead of using real-world sound samples or physical model-based sounds is useful as it avoids any confusion with ambient sounds. In addition, the sounds are selected to avoid interference with speech/vocal interaction. The pitches are in a middle/upper range of musical frequencies, where the sensitivity is high and low-cost loudspeakers easily project the sounds over several meters.
**Backgrounding and Saliency:** The sound streams should be regular (e.g., in rhythmic and temporal organization) to facilitate listeners’ habituation to these sound streams. Habituation is a perceptual skill allowing one to ignore sound streams (in favor of other tasks or sound streams) yet to remain sensitive to relevant changes in those background streams. An example is the sound of the refrigerator in the kitchen, or the exhaust in out-dated fossil-fuel-driven automobiles, which drivers clearly hear but can ignore until their sound pattern changes due to malfunction. However, mapping absolute ST values to more extreme pitches and sharpness, changes can be expected to be salient enough to draw the listeners’ attention.
**Universality:** Musical sounds are perceived according to the cultural background. The current choice of intervals, however, is largely driven by consonance, which is a more universal feature: the octave is a preferred interval in most musical communities, and the fifth and fourth are generally preferred consonant intervals.

Based on these criteria, we proceeded with design selection and method fine-tuning. We have tested multiple variants, including how it would appear at various realistic heart rates.

Concerning the channel model, we decided to play limb leads counterclockwise and to negate (invert) lead aVR so that the series becomes aVL, I, −aVR, II, aVF, III as depicted in the lower panel of Figure 4 and Figure 6. The clinical reason for this procedure is that by doing so, we have the ST elevations of the limb leads clustered. The resulting channel mode is the sequence of limb leads counterclockwise and precordial leads counterclockwise (viewed from the top downward).

Regarding the temporal organization (i.e., set separation and stride), the clinicians rated dt=0.12 as a lower limit—since they believed that students otherwise could not properly resolve the channels. The slower version was regarded as being easier to learn and to comprehend, particularly within the scope of a study that leaves little time to familiarize. From a perspective of musical structures where patterns often take 4, 8 or 16 bars, a stride of eight heartbeats between repetitions of the sonification was subjectively preferred. To obtain a clear separation of the limb and precordial lead groups, a separation of two heartbeats is the minimum, i.e., limb leads/augmented leads played after the first beat, precordial leads starting with the third beat.

Regarding the pitch mapping, the tristate appeared to be ‘too coarse’ and did provide enough detail. On the contrary, continuous mapping and chromatic mapping offered too much variability to be processed with manageable effort. Further, it appeared to have worse aesthetics with many intervals that did not fit. This directed our attention towards the five-state sonifications, which were preferred by the clinicians, taking into account their expertise with medical alerts, soundscapes in the operating theater, and emergency situations. The solution was regarded as offering the highest discernibility and the lowest risk of alarm fatigue. If non-urgent or even false alarms are repeatedly detected by nurses or other staff, alarm fatigue may be the result, and staff might not react in the proper time if genuine emergency alarms are sounding [29]. The five-state dissonant mapping was regarded as compelling concerning the quality of sound: dissonant sounds would have a stronger call for users’ actions to resolve a precarious situation. However, if a five-minute or even longer journey to the hospital is involved, then the constant need for the emergency crew to listen attentively to dissonant sounds while not being able to do anything about it will probably be pointless, which is why we eventually opted for the more pleasant/consonant five-state mapping.

In summary, for the study described in the following section, our sonification method assumes a QRS tone frequency of $f_{0}=554$ Hz and a pulse rate of 80 bpm, along with the following parameters: With respect to a tone’s fundamental frequency, we use (i) eight semitones below $f_{0}$ for a strongly suppressed ST (below −0.2 mV); (ii) five semitones below $f_{0}$ for a moderately suppressed ST (i.e., in [−0.2 mV, −0.1 mV]); (iii) $f_{0}$ for the non-elevated case (i.e., in [−0.1 mV, 0.1 mV]); (iv) four semitones above $f_{0}$ for a moderately elevated segment (in [0.1 mV, 0.2 mV]); and (v) seven semitones above $f_{0}$ for a strongly elevated ST segment (above 0.2 mV). Thereby, a tone’s frequency for any given reference frequency $f_{0}$ and semitone offset *k* is computed by $\mathrm{frequency}\left(k\right)=f_{0}\cdot 2^{k/12}$.

The time difference between ST-tone onsets is dt=120 ms. The set separation is 8, i.e., a new sonification starts after every eight heartbeats. The stride is 2; i.e., the limb lead is anchored to the first beat, whereas the precordial lead set is anchored to the third (=1 + stride)th heartbeat. The duration *d* of notes is 50 ms (respectively, 80 ms, 100 ms) for absolute values <0.1 mV (resp. [0.1 mV, 0.2 mV], >0.2 mV). The tone level *L* is subject to individual adjustment; however, we apply a 5 dB boost for absolute values >0.1 mV (respectively, 15 dB for |st|>0.2 mV). This offset itself is scaled by a parameter dBscale so that a tone’s actual level is the global base level $L_{0}+\mathrm{dBscale}\cdot L$. We chose dBscale =0.2 and $L_{0}=-25$ dB. The tone’s sound signal itself is $s\left(t\right)=\frac{1}{N}\sum_{n=1}^{N}\cos\left(2\pi nf_{0}-n\pi /2\right)$, i.e., a sum of harmonics, appropriately phase-shifted to align the peaks. N=2 for |st|<0.1 mV, else N=4. We set the amplitude envelope as $w_{c,d}\left(t\right)=1-\left(1-e^{c\cdot t/d}\right)/\left(1-e^{c}\right)$ where *c* is the curve, *d* the tone’s duration, and *t* the time, in order to control how the tones fade out. We set c=3 for absolute values |st|>0.1 mV (respectively, −3 for absolute values |st|<0.1 mV) to make iso-electric ST tones appear more percussive. We provide an implementation of this sonification in Python as Supplementary Materials. This contains a function that takes a 12-lead vector of ST elevations as input and generates both a wave file and a visual plot of the sonification for reproducing our stimuli.

**Figure 10:**
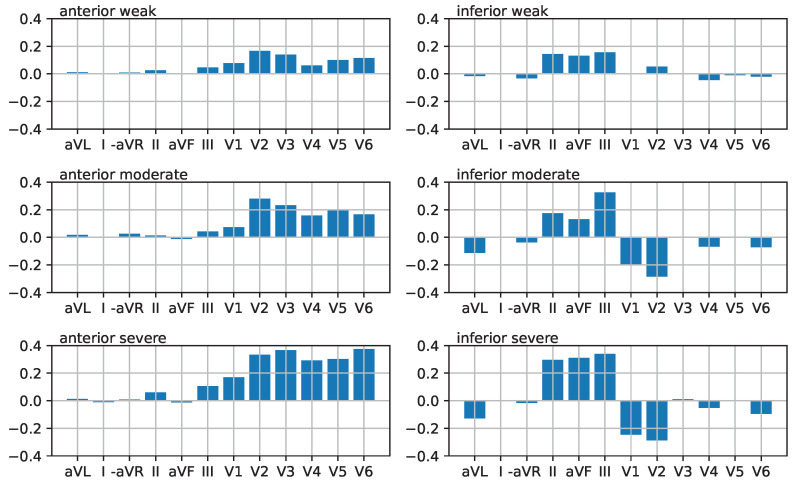
ST elevation plots: left: anterior STEMI, right: inferior STEMI, from top to bottom: weak, moderate, severe. Data corresponding to the six cases derived from Laerdal simulator data as explained in Section 3 and shown in Figure 4.

For the classification study described in Section 6, we used the ST elevation profiles depicted in Figure 10 obtained from the Laerdal simulator, as detailed in Section 3. The same data are shown as a parallel coordinate plot in Figure 4. These plots helped subjects understand the data and sonification for the classification study.

## 6. Classification Study Design

### 6.1. Overview

The local ethics committee of the Ruhr University Bochum, situated in Bad Oeynhausen, Germany, gave approval (file no.: 2022-1017). The study followed the principles of Helsinki.

The entire study consisted of a perception and STEMI classification study using audibly presented (sonified) ECG sequences, as well as a scenario study in the form of a randomized controlled trial (RCT) in order to compare sonification, assisted with a standard visual-based diagnostic process in an emergency situation. Here, we focus on the perception and classification part, whereas the results of the scenario study will be published elsewhere.

The long-term goal of the entire study is to enable and establish sonification in the medical emergency service. Therefore, the most important guiding principle here was to define a patient-relevant endpoint for an RCT. With regard to the emergency case, the correct classification of STEMI is merely an intermediate step, which presumably turns out differently in stressful situations versus a purely stress-free exercise situation. A patient-relevant endpoint is, therefore, the timely admission of a patient to the catheterization laboratory. Here, we refrain from elaborating on this endpoint and instead postpone the presentation of the corresponding RCT to a forthcoming article (for a conference abstract, see [7]).

Of note, in order to ethically justify a study in an emergency setting, a preliminary study with a positive result and the design of a fallback strategy are absolutely necessary. An integration of this study concept into the emergency training of medical students therefore seemed predestined to create a scenario that is as real as possible and is located outside the real emergency operation. At the same time, a teaching concept is being developed. Part of this teaching concept is the introduction to sonification and the estimation of diagnostic accuracy in a first classification step, as presented here. The introduction to sonification and this first classification step thus represent the first module of a bi-modular approach. We refrained from a comparative study in the first module because both the pure classification quality without the temporal aspect is clinically not very relevant in stressful situations but also because the cohort size would not have permitted a further study arm in the first module.

Although no control group was planned for this first module, the study nevertheless serves to address the accuracy of the diagnosis as a function of various factors that are ultimately essential for the further development of the sonification design. The performed regressions therefore introduce a semi-quantitative aspect. As a final note, we would like to point out that we are aiming for sonification to optimize emergency operations; i.e., instead of addressing professional cardiologists, we are rather planning to train emergency paramedics, which should once again make the study setting in the context of emergency training plausible. Finally, cardiology experts also participated in the expert evaluation committee to select the best sonification method.

Further, basic epidemiological parameters were collected, and questionnaire-based surveys were carried out. A first survey regarding score-based self-assessments of previous experience with emergency situations and musical expertise, as well as questions concerning the general attitude toward sonification, took place before the study was carried out. Specifically, we asked forthe subjects’ agreement with the following statements on a Likert scale from 1 (not at all) to 5 (completely):

Q_1_ I had experience with pre-clinical emergencies.
Q_2_ I have participated to more than 3 emergency trainings.
Q_3_ I feel confident when handling emergency situations.
Q_4_ For me, emergency situations cause negative stress.

After the perception test, we asked for the subjects’ agreement with the following three statements:

SQ_1_ Sonification is pleasant to listen to
SQ_2_ The sonification is informative, i.e., it enables to identify ST elevation changes in the ECG
SQ_3_ I can imagine to listen to these sonifications for a longer time period

### 6.2. Participants

After receiving the certificate of nonobjection from the local ethics committee on 20 January 2023, data acquisition began, which ended on 30 June 2023. The study cohort consisted of 44 students from the faculty of medicine of the Ruhr University Bochum (RUB) in Germany (5th academic year, corresponding to 9th and 10th semesters), who were assigned to a three-day training program in emergency medicine. After being informed about the nature, significance, and scope of the study, as well as the requirements for participants, but prior to the study, participants provided informed consent in written form by signing a declaration of consent, and also consented to the processing of study data. All students received a 10 min review about diagnosing and treating STEMI in prehospital care. Since the sonification of the ST segment was a new concept, they received an introduction to sonification, particularly ECG sonification. For this purpose, all participants received a 6:15 min introduction video about sonification, its impact on ECG monitoring, and the motivation of our research. As part of the introductio, n we presented audio examples of ECGs either in isoelectric mode or with significant elevation of the ST segment to the students.

Before starting the perception and classification study, we asked the participants to answer an initial questionnaire for the collection of basic demographic data (i.e., age, gender, medical education) and for experience in medical emergencies. Since it could affect the comprehension of sonification, they were also asked their pre-existing experience in music (see Supplementary Materials for the complete questionnaire). Summary statistics depicting the composition of the cohort stratified by gender, including the self-assessment scores derived from the survey and the assignment to either the intervention or the control group of an RCT (to be published elsewhere) i.e., parameter sonification, are shown in Table 1.

### 6.3. Classification Task

The sonification used in this study was designed for the detection of an ST segment elevation in the ECG that fulfills the STEMI criteria according to ESC guidelines (see 3.1 for details). A total of three similar blocks of five distinct sonified ECG-variants (IE, anterior–moderate, anterior–severe, inferior–moderate, inferior–severe) were presented to all of the participants (n=44) leading to 3×5×44=660 classification instances. Participants were asked to classify the samples with regard to the localization (anterior or inferior) and severity (IE, moderate or severe) of the ST elevation using the questionnaire mentioned before.

## 7. Statistical Methods

Sonifications of 15 ECG examples were presented to 44 individuals with the task of classifying the five different STEMI types, leading to 15×44=660 classification instances. The classification performance is evaluated on the basis of a 5×5-confusion matrix depicting the frequencies of the 660 classification results versus the correct classifications (gold standard) for each of the five classes. The estimation of diagnostic parameters, particularly accuracy, sensitivity, and specificity, is based on the confusion matrix. The nested structure of the classes (grouping into one normal ECG and two cases each of moderate and severe STEMI, as well as the decomposition into successively presented samples) is accounted for by sub-matrix analyses. Quantitative results are reported as overall accuracy, sensitivity (se), specificity (sp), F-score (f1=se·sp/(se+sp)), balanced accuracy per class (ba=(se+sp)/2), and intraclass coefficient κ [30].

A multivariable linear random effects regression allows quantification of the effects of covariates (demographic parameters, self-assessments and pre-knowledge, and index of the chronological order of ECG presentation) on the classification performance. The random effects model accounts for the repeated measurement structure of the trial by adding the person-ID as a random effect [31]. Univariable two-sample comparisons are based on *t*-tests for metric data and Fisher’s exact test or chi-squared test for categorical data. The chosen significance level was 5%, and precision is reported using 95% confidence intervals.

Statistical analyses were performed with R (version 4.3.2) [32].

## 8. Results

Summary statistics containing age and scores of self-assessment derived from questionnaires stratified by gender are shown in Table 1. In total, more female (*n* = 31) than male (*n* = 13) students participated in the study. Female students were slightly younger on average.

### 8.1. Excellent Performance of Sonification-Assisted STEMI Classifications

The results of the 660 classification instances are depicted as a confusion matrix in Table 2.

The ECGs without abnormality (IE),three samples of which were presented to each of the 44 students for classification, were all correctly classified as ‘healthy.’ In some instances, the severity of STEMI was falsely classified; however, there is only one instance where a moderate inferior STEMI (I-moderate) was been diagnosed as ‘healthy’ (IE). In addition, occasionally an ‘ST elevation inferior’ was confused with ‘ST elevation anterior’ and vice versa; however, the overall classification performance turned out to be excellent. Overall accuracy was 0.82 (0.79, 0.85), and intraclass coefficient turned out to be κ=0.77. Class-specific diagnostic parameters are listed in Table 3.

In a dichotomized version of the classification problem, IE is coded as 0 (‘healthy’), whereas all other classes are combined into one positive class coded as 1, i.e., STEMI independently of severity and location. This results in the confusion matrix depicted in Table 4. Remarkably, all 660 classification instances yielded a perfect classification with an accuracy of 1 (0.99, 1) and both a sensitivity and a specificity of exactly 1.

Finally, we combined the two severe and the two moderate classes into one class each, independently of location (inferior or anterior). This gives rise to the confusion matrix depicted in Table 5.

As the most important adverse result, 29 severe cases were predicted as moderate. The corresponding estimated class-specific diagnostic test parameters are listed in Table 6.

Overall accuracy was estimated as 0.91 (0.88, 0.93) and κ=0.86.

Thus far, we find that discrimination of IE from STEMI classes yields a perfect result with sensitivity and specificity both equal to 1. Although not perfect, sonification-assisted differential diagnostic performance within the STEMI classes is still hugely satisfying, with an overall accuracy of 0.82 and with 0.91 even larger, if the location is disregarded and only severity considered.

### 8.2. Uncertainty in Coping with Emergency Situations Affects Sonification-Based Classification Performance

In an alternative way, accuracy per class can be estimated on the basis of a linear model after numeric 0-1 coding of the outcome per classification instance (1 if correctly classified, 0 otherwise). This approach can straightforwardly be extended to multivariable random effect linear regression in order to assess the impact of demographic determinants or scores from self-assessment, like prior knowledge and uncertainty in coping with emergency situations, in the classification performance. In this case, correlations due to repeated measures can be adequately handled by adding the person index as a random variable. The corresponding regression results are shown in Table 7.

Results for the class indicators are not shown since they only function as dummy variables. Reference levels for categorical predictors, as well as the intercept, are also omitted.

Gender, scores Q3 and SQ2, and block 3 turn out to have a statistically significant impact as predictors for the outcome of the sonification-assisted ECG classification task. Specifically, female students perform significantly better than male students. Q3 refers to question 3 of the first questionnaire, where each student was asked whether they feel confident when handling emergency situations. It is worth noting that we observed a counterintuitive negative effect. Apparently, less confidence correlates with better classification. Interpretations, however, particularly causal inferences, have to be drawn with utmost care. More intuitive is the result with respect to an increased classification performance for block 3. This variable refers to the last of three consecutively presented blocks of sonification samples, thus pointing to a learning effect over the course of time through repetitions. Finally, the score derived from question SQ2—which asked, after students finished the sonification-assisted classification rates in ascending order, whether the students regarded sonification to be informative—correlates positively with classification accuracy. The remaining ratings have negligible impact. For the sake of completeness, variances of the random effects turn out to be 0.016 (intercept) and 0.11 (residual). Thus far, we conclude that some self-assessment scores correlate in an intuitive way with the outcome, with the exception of Q3. Roughly speaking, feeling more or less comfortable with sonifications improves classification performance; however, prior experience with emergency situations appears to be counter-productive. Also somewhat surprising is the fact that females perform better than males with respect to correctly classifying sonification-assisted ECG samples.

## 9. Discussion

A first and primary focus of this article was the sonification concept, design, and implementation of the ST elevation sonification method. The step-by-step development process from ‘heartbeat-locked ST elevation arpeggio’ over ‘musical phrase melody over several heartbeats’ to ‘grouped lead scans’ illustrated how, step-by-step, sonification design progresses toward a solution that addresses issues such as cognitive load, attention, annoyance, perceptability and ecological acoustics, i.e., how sonifications integrate into the soundscape. We acknowledge that choices within the design progression are made on the basis of subjective decisions and not on objectively derived evidence. However, as in any other field of design, such as industrial or visual design, a fully objective evidence is not feasible. Retrospectively, however, usability or, in the given context, diagnostic value is objectively testable, the way we used it here. Further results will be published soon.

The design of sonification was initially based on the assumption that conveying as much information as possible in a timely manner was best. The ‘heartbeat-locked ST elevation arpeggio’ conveys all 12-lead elevations with each heartbeat. This could be useful if, e.g., medical interventions would require a precise and low-latency eyes-free monitoring of these details, for instance, for balloon-catheter interventions or intermittent pre-clinical ST elevation representations in the ECG (e.g., occlusive myocardial infarction). It is noteworthy that the sound level could be considerably lowered in practical applications; i.e., we expect the sound to become part of the *available periphery*, as in Mark Weiser’s vision of calm computing [33], an information stream that is available yet only attracting attention when needed—and thereby reducing distraction.

We realized that for extended monitoring (e.g., over many minutes to hours) the initially chosen amount of detail is overwhelming. Additionally, ST patterns change rarely between beats. Therefore, we explored a complementary approach in design 2 (musical phrase melody over several heartbeats), which reduces information density. However, a specific melody repeated many times over hours could be perceived as intrusive, and would also conflict with situations in which music is heard. Design 2 could be of interest for applications in long-term monitoring, particularly if it were modified by fading out upon absent changes and by boosting the sound level upon relevant ST changes over time.

Design 3 (the grouped lead scans) represents a compromise between precision and aesthetics, between low-latency and acceptable delay, in such a way that grouping is supported, thus revealing auditory gestalts (for typical motifs corresponding to clinically relevant parts).

All designs use *pitched tones*, as these are clearly recognized as artificial, to avoid the risk of confusing the auditory display with environmental sounds. Alternative ideas, such as water drop sounds and footstep sounds, have been dropped. However, it might be worth investigating other pitched real-world sounds as material, as these could lead to higher acceptance and reduced annoyance. We put this on our roadmap for future optimization.

We envision integrating sonification into commercial ECG devices. The computational requirements are rather low for the mapping and sonification rendering. For sound projection, due to the artificial nature of the sound, small loudspeakers would suffice, which could easily be integrated in devices.

It will be important that personnel have direct control to (de)activate sounds and control their sound levels. A more futuristic perspective could be the integration of monitoring sounds into the personal soundscape of medical staff, e.g., via transparent earplugs that do not block external sound but leave patients and others who do not need to be informed in a calm state.

In emergency situations, sonification may lead to cognitive overload, which needs evaluation. In the case the two groups of six sound events prove to be too much, the following pathways reduce the risk of cognitive load by condensing the information: (i) by merging two or three tones into one, in turn decreasing the spatial resolution of the sonification while maintaining the logic of suppression/elevation being represented as musical intervals, or (ii) by an automatic saliency control that couples the sound level of these additional elements to the amount of difference from the ST elevation profile of the initially recorded ECG.

In the advent of clinical AI-based diagnosis and information systems, it might appear questionable whether the proposed rather direct/low-level information approach would be superior to an AI method that condenses data and directly communicates the essential information to users. Recent approaches in replacing diagnostics completely by using AI and deep learning show high potential, as is the case, for instance, for cardiac arrhythmia diagnosis [34] or abnormality classification of beat features [35] and also STEMI classification [36]. However, since we target scenarios where the aim is to remain sensitive to gradual changes in ST elevations, which may emerge, e.g., during an emergency transport, sonification has the benefit of being an analog representation, which is preferred in such situations. This is comparable to the pitch modulation used in a pulse oximeter to detect gradual changes in oxygen saturation. Furthermore, AI-based approaches bear the risk of false positives and go along with an interruption of other ongoing processes (in an emergency situation), while the low-level monitoring continuously supplies information and leaves the evaluation to the human. Human operators usually feel more in control if they perceive continuous changes or the actual state as compared to being informed only of critical changes.

We used surrogate ECG data obtained from the Laerdal Resusci Anne Advanced SkillTrainer^®^. We observed little variability in the measured data over time and obtained rather stable ST elevation patterns over time. The next step would be to apply the chosen design to real-world data from emergency procedures. This data will include various artifacts such as shivering, interference from other electronic devices, body movements, loose electrodes, or problems due to incorrect lead placement. Thus, the question becomes how increased variability in ST elevation sonification would be interpreted and to what extent medical conclusions depend on these factors. For the present study, however, the limitation of using the Laerdal System is outweighed by the advantage that a clear and reproducible gold standard is defined. The classification carried out is therefore significantly less susceptible to bias than would be the case with a gold standard defined by experts in the case of real data.

This study reveals that ST elevation, a pathology of the ECG, can reliably be detected by a defined minimally trained cohort of medical students. The data were transferred to a novel specially designed ST elevation sonification of the 12-lead ECG. ST elevation is the most important part of the ECG when assessing patients with chest pain and therefore essential for diagnosing STEMI [3]. After a short training period, subjects demonstrated good recognition of the different sounds, suggesting that more precise discrimination could be achieved with longer training. A 6.5-minute video tutorial was sufficient to teach the method to subjects who were naïve to sonification.

Training is known to improve pattern recognition in regular visual ECG learning [37]. Therefore, the same is to be expected for ECG sonification of the ST segment.

We investigated a group of medically trained individuals. However, among this otherwise quite homogeneous group, we observed structural variance. Demographic covariates include the fact that some of the students had worked in EMS previously, and some had musical training. However, the proposed method is designed to be generally suitable for EMS, and even in hospital setting, this pre-trained group of test subjects appears to be representative.

The use of sonification to improve the correct interpretation of abnormal ECG data and/or signals has already been tested in several preliminary studies. In 2017, Kather et al. [20] showed that the auditory presentation of ECG data can enable the detection of clinically relevant cardiac pathologies (including bigemini, atrial fibrillation, or STEMI) by non-medically trained volunteers and after a short training session. Additionally, this study demonstrated that a cohort of 5th-year medical students had been able to correctly identify the severity and type of STEMI with a high degree of sensitivity.

Sonification of the ST segment has been scientifically investigated in previous studies [4,5], and the potential for detecting ST elevations has already been demonstrated. ST elevations can be transient and therefore not detectable at all times. Since this ECG pattern in the context of a myocardial infarction results in a different treatment priority, it is crucial to detect it. Sonification is a helpful method for detecting these transient ST elevations in this constellation.Additionally, different degrees of ST-segment elevation could be detected. It is to be expected that the just noticeable differences (JNDs) could become smaller with increased training.

Compared to realistic settings, the perception study has the limitation that the participants were not distracted by other impressions, particularly not by impressions of auditory nature. This limits the generalization power. In the future, users can be taught this method by using tutorials as video clips. It is not practical to replace the current practice of printing out a 12-lead ECG or visualizing it in another way. However, ST elevation sonification can be a valuable add-on to current practice, as sonification can draw attention to transient ST elevations.

## 10. Conclusions

We introduced a variety of sonification designs for monitoring ST elevation patterns according to their intensity, polarity, and spatial distribution, which is connected to relevant detail corresponding to specific heart pathologies (coronary occlusion). We chose design 3 (grouped lead scans) for EMS as a trade-off between distraction and timely, precise, and detailed information. The discretization of continuous ST elevations into five levels reduces auditory variability while maintaining enough information for users to distinguish states. The ST segment of an ECG can be sonified to be perceived as pleasant.

A qualitative assessment and descriptive statistics of the classification process show that ST changes can be detected reliably, and the procedure can be learned easily and in a short period of time. In emergency situations, using sonification has the potential for the surveillance of the ST segment with high sensitivity and specifity to detect changes. Specifically, the classification, which was carried out as part of a student training program for rescue personnel, prepares an RCT in the context of a simulation of a rescue situation with the same test subjects. This means that the study group corresponds exactly to the group whose emergency service skills are to be enhanced by sonification. ST segment sonification enables the *continuous* process monitoring for intermittent ST segment elevations so that they can be identified promptly. This auditory display method is a promising auxiliary help for diagnosing ST elevation myocardial infarction, leading to immediate consequences in treatment.

The possible clinical application of ST segment sonification can be in EMS, the emergency room, and the catheterization laboratory. Future studies should examine, as part of a proof-of-concept, whether the results achieved in the presented study also have a corresponding impact after translation into clinical practice.

## Figures and Tables

**Table 1:** Summary statistics of self-assessment scores, demographic parameters, and group assignment stratified by gender. Q1–Q4 and SQ1–SQ3 refer to the self-assessment scores collected by means of questionnaires. “Preknowledge” refers to previous medical training, whereas “Musicality” and “Instrument” refer to single-choice formal information on musical education. For details on questionnaires, cf. the Methods section.

Characteristic	Female, N=31	Male, N=13	*p*-Value
Age	23.00 (23.00, 24.00)	24.00 (23.00, 25.00)	0.2
Q1	4.00 (3.00, 5.00)	4.00 (4.00, 5.00)	0.9
Q2	4.00 (2.00, 5.00)	4.00 (2.00, 4.00)	0.6
Q3	4.00 (3.00, 4.00)	4.00 (3.00, 4.00)	0.6
Q4	2.00 (2.00, 3.00)	3.00 (2.00, 3.00)	0.2
SQ1	4.00 (3.00, 5.00)	4.00 (3.00, 5.00)	0.5
SQ2	5.00 (4.00, 5.00)	4.00 (3.00, 5.00)	0.4
SQ3	3.00 (2.00, 4.00)	3.00 (2.00, 5.00)	0.9
Preknowledge	3/31 (9.7%)	1/13 (7.7%)	>0.9
Musicality	2/31 (6.5%)	1/13 (7.7%)	>0.9
Instrument	14/31 (45%)	7/13 (54%)	0.6

n/N (%) Median (IQR); Fisher’s exact test; Welch two-sample *t*-test; Pearson’s Chi-squared test.

**Table 2:** Confusion matrix depicting the classification frequencies of predicted classes (Prediction) versus the correct classes (Gold Standard).

	Gold Standard					
	IE	I-moderate	I-severe	A-moderate	A-severe	Total
**Prediction**						
IE	132	0	0	0	0	132
I-moderate	0	106	14	15	0	135
I-severe	0	8	95	4	13	120
A-moderate	0	12	4	100	11	127
A-severe	0	6	19	13	108	146
Total	132	132	132	132	132	660

**Table 3:** Estimated class-specific diagnostic parameters of the sonification-assisted STEMI classification task.

Class	Sensitivity	Specificity	f1	Balanced Accuracy
IE	1	1	1	1
I-moderate	0.8	0.95	0.79	0.87
I-severe	0.72	0.95	0.75	0.84
A-moderate	0.76	0.95	0.77	0.85
A-severe	0.82	0.93	0.78	0.87

**Table 4:** Confusion matrix for the dichotomized classification problem.

	Gold Standard		
	0	1	Total
**Prediction**			
0	132	0	132
1	0	528	528
Total	132	528	660

**Table 5:** Confusion matrix for the reduced classification task with combined classes corresponding to moderate and severe cases, respectively, independently of location.

	Gold Standard			
	IE	moderate	severe	Total
**Prediction**				
IE	132	0	0	132
moderate	0	233	29	262
severe	0	31	235	266
Total	132	264	264	660

**Table 6:** Estimated class-specific diagnostic parameters of the sonification-assisted STEMI classification task with the two severe and the two moderate classes each combined to a single class irrespective of location.

Class	Sensitivity	Specificity	f1	Balanced Accuracy
IE	1	1	1	1
moderate	0.88	0.93	0.89	0.9
severe	0.89	0.92	0.89	0.9

**Table 7:** Fixed-effect regression results from a multivariable linear random effects model, which regresses the classification result (prediction) on 13 demographic and self-assessment parameters (predictors). Reference levels for categorical variables are omitted. “Block” refers to the iteration of consecutively presented blocks of sonification samples. For explanations of the other covariates, see Table 1 and the Methods section, respectively.

Predictors	Estimate	Std.Error	*p*-Value
Gender female	0.18	0.06	0.005
Age	−0.001	0.018	0.968
Q1	0.034	0.04	0.399
Q2	0.024	0.031	0.442
Q3	−0.115	0.042	0.01
Q4	0.057	0.039	0.158
Preknowledge yes	0.087	0.149	0.562
Instrument yes	0.012	0.06	0.846
Musicality yes	0.06	0.107	0.578
SQ1	−0.011	0.024	0.663
SQ2	0.053	0.021	0.015
SQ3	0.011	0.023	0.653
Block 2	0.05	0.032	0.114
Block 3	0.091	0.032	0.004

## Data Availability

All relevant processed data are within the manuscript and its Supplementary Files. Raw ECG data cannot be publicly shared due to legal reasons.

## Supplementary Materials

The following supporting information can be downloaded at https://www.mdpi.com/article/10.3390/s25144373/s1. Sonification Examples for Design 1: The supplementary material contains four MP3 files S1-1-a-all-six-conditions.mp3, S1-1-b-all-six-conditions.mp3, S1-2-percussion-all-six-conditions.mp3, S1-3-vowels-all-six-conditions.mp3.Sonification Examples for Design 2: The supplementary material contains 6 MP3 files, one for each STEMI condition: S2-musicalphrase-inferior-weak.mp3, S2-musicalphrase-inferior-moderate.mp3, S2-musicalphrase-inferior-severe.mp3, S2-musicalphrase-anterior-weak.mp3, S2-musicalphrase-anterior-moderate.mp3, S2-musicalphrase-anterior-severe.mp3. Sonification Examples for Design 3: The supplementary material contains 34 MP3 files. The first 30 sonification examples (S3.01-S3.30) are in groups of six files (one for each STEMI condition) according to the five modes ‘chromatic’, ‘diatonic’, ‘5- state’, ‘5-state-dissonant’, and ‘tristate’. The remaining four sonification examples (S3.31–34) illustrate fast mappings, suitable for trained listeners, cf. the file names for details on the parameters; Source Code (Python) for Design 3 (Grouped Lead Scans): The supplementary material contains a Python Jupyter notebook that implements the algorithm for rendering sonifications of our reference ST elevation profiles or custom profiles.

## Abbreviations

The following abbreviations are used in this manuscript:

ECG	Electrocardiography
EMSs	Emergency Medical Services
PMSon	Parameter Mapping Sonification
STEMI	ST elevation Mycardial Infarction
