# Supplementary material for: ST Elevation Sonification of a 12-Lead ECG for the Assessment, Diagnosis, and Monitoring of ST Elevation Myocardial Infarction
Source: Sensors (Basel). 2025 Jul 12;25(14):4373. doi: 10.3390/s25144373 (PMC12299781; doi:10.3390/s25144373)
Supplement: Supplementary file 1 [file sensors-25-04373-s001.zip › supplementary-material/S4-SourceCode-Python/STElevationSonification-GroupLeadScans-5state-mapping.pdf]

STElevationSonification-GroupLeadScans-5state-mapping

July 11, 2025

0.1 Supplementary Material: Python code to render final sonification

- by Thomas Hermann, 2025-06-25
- Source code as supplementary material for the article "ST Elevation Sonification of 12 lead ECG for Assessment, Diagnosis and Monitoring of ST Elevation Myocardial Infarction"

- This notebook contains an implementation of the Grouped Lead Scans Sonification method.
- The implemented method is the 5-state mapping, which was used in the study.
- Note that the notebook uses pya `https://pub.uni-bielefeld.de/record/2938411`, a Python Library for Audio Processing and Auditory Display
  - available at https://github.com/interactive-sonification/pya
- Note that in addition pya Agens `https://pub.uni-bielefeld.de/record/3003417` are used.
  - as of June 2025, installation of the feature-agen branch from pya is required to use these generators. It is planned to include these features into the next release of pya, so it may be available via `pip install pya` in/after October 2025.

[1]:
```
from pya import Asig, startup
from pya.agen.lib import BLImp, Line

import matplotlib.pyplot as plt
import numpy as np

import pyamapping as pam

s = startup()
```

Prepare data for all conditions:

- isoelectric
- anterior moderate
- anterior severe
- inferior moderate
- inferior severe

For each we need the clockwise cabrera set and the precordial set in the order and polarity used in the paper

[2]:
```
sts_precordial_dict = {
    "anterior_moderate": np.array(
```

```
        [0.07203159, 0.2794078, 0.23152477, 0.15677434, 0.19189014, 0.16524862]
    ),
    "anterior_severe": np.array(
        [0.17095923, 0.3329215, 0.36629564, 0.29230115, 0.30242586, 0.37456089]
    ),
    "inferior_moderate": np.array(
        [-0.1947353, -0.28563085, 0.00180213, -0.06779367, -0.0004029, -0.
 ↪07203096]
    ),
    "inferior_severe": np.array(
        [-0.24575369, -0.2876513, 0.01108831, -0.05076629, -0.00420424, -0.
 ↪09491774]
    ),
    "IE": [0, 0, 0, 0, 0, 0],
}

sts_cabrera_dict = {
    "anterior_moderate": np.array(
        [0.01694881, -0.00423711, 0.02542282, 0.01271115, -0.01271168, 0.
 ↪04237111]
    ),
    "anterior_severe": np.array(
        [0.01339769, -0.00973679, 0.00885468, 0.05931953, -0.01243168, 0.
 ↪1059798]
    ),
    "inferior_moderate": np.array(
        [
            -1.13693699e-01,
            -2.75322702e-04,
            -3.65873910e-02,
            1.73722625e-01,
            1.31350726e-01,
            3.26259583e-01,
        ]
    ),
    "inferior_severe": np.array(
        [-0.12711412, 0.0042374, -0.01711861, 0.29659986, 0.3107805, 0.33941242]
    ),
    "IE": [0, 0, 0, 0, 0, 0],
}
```

[3]:

```
plt.figure(figsize=(9, 3))
cases = sts_cabrera_dict.keys()
for i, case in enumerate(cases):
    plt.plot(
        np.arange(6),
        sts_cabrera_dict[case],
```

```
        label=case,
        color=["r", "g", "b", "c", "m", "k"][i],
        marker="o",
    )
    plt.plot(
        np.arange(6) + 6,
        sts_precordial_dict[case],
        color=["r", "g", "b", "c", "m", "k"][i],
        marker="o",
    )
plt.legend(loc="upper left", fontsize=7)
plt.grid()
plt.ylabel("ST elevation [mV]")
plt.xticks(
    np.arange(12),
    ["aVL", "I", "-aVR", "II", "aVF", "III", "V1", "V2", "V3", "V4", "V5",␣
 ↪"V6"],
);
```

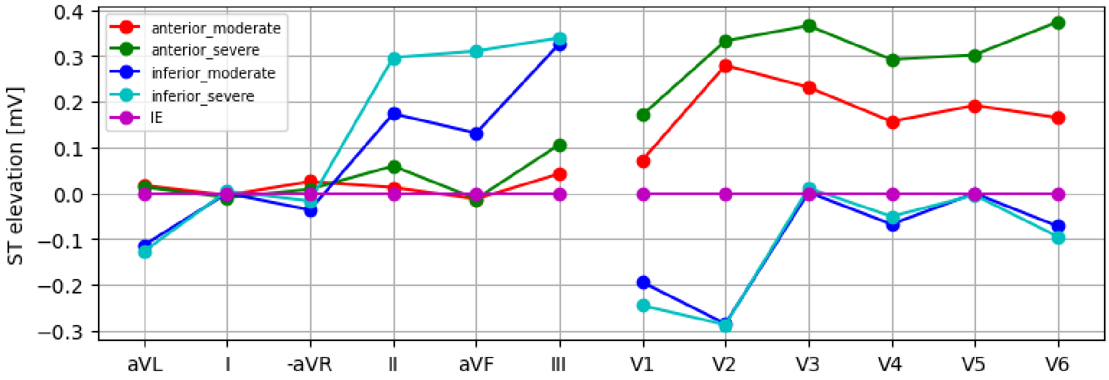

[4]:
```
# load qrs tone
aqrs = Asig("sounds/QRSTone.wav").resample(44100)
aqrs.gain(0.5).play()
```

[4]:
```
Asig('sounds/QRSTone.wav_resampled_scaled'): 1 x 7497 @ 44100Hz = 0.170s
cn=['0']
```

[5]:
```
def lead_event(freq=554, amp=0.1, num=1, dur=0.05, curve=2):
    "Generator for a single ECG lead tone. Returns an Asig."
    ag1 = BLImp(freq, numharm=num) * Line(1, 0, dur, curve=curve) * amp
    return ag1.gen_asig()


def lead_group(sts=[0, 0, 0, 0, 0, 0], dt=0.12, f0=554, level=-10, dbscale=0.2):
```

```python
    "Render a set of lead events for the vector sts of st elevations into Asig."
    acanvas = Asig(1.0)
    for k, x in enumerate(sts):  # x = ST elevation
        # mapping to frequency
        if x < -0.2:
            semitones = -8
        if -0.2 < x < 0.1:
            semitones = -5
        if -0.1 < x < 0.1:
            semitones = 0
        if 0.1 < x < 0.2:
            semitones = 4
        if 0.2 < x:
            semitones = 7
        freq = f0 * 2 ** (semitones / 12)

        # mapping to duration
        tone_duration = 0.05 if abs(x) < 0.1 else 0.08 if abs(x) < 0.2 else 0.1

        # mapping to brightness
        numharm = 2 if abs(x) < 0.1 else 4

        # mapping to amplitude
        lvl = (
            level
            if abs(x) < 0.1
            else level + 10 * dbscale if abs(x) < 0.2 else level + 15 * dbscale
        )
        amp = pam.db_to_amp(lvl)

        # mapping for amplitude envelope (curvature)
        curve = -3 if abs(x) < 0.1 else 3

        # render sound
        asig = lead_event(
            freq=freq, amp=amp, num=numharm, dur=tone_duration, curve=curve
        )
        # add sound to canvas
        acanvas.x[{(k + 1) * dt: None}] += asig
    return acanvas


def render_ST_elevation_sonification_example(
    st_cabrera=[0, 0, 0, 0, 0, 0],
    st_precordial=[0, 0, 0, 0, 0, 0],
    dt=0.120,
    set_separation=8,
```

```
    stride=2,
    bpm=80,
    nrep=1,
    level=-25,
):
    """Render the 5-state mapping Lead Group Scan sonification.
    Parameters are described in the article.
    Returns the sonification as Asig.
    """
    onset = 0
    acanvas = Asig(1.0)
    for j in range(nrep):
        for i in range(set_separation):
            acanvas.x[{onset: None}] += 0.3 * aqrs
            if i == 0:  # generate limb leads sequence
                ar = lead_group(sts=st_cabrera, dt=dt, level=level)
                acanvas.x[{onset: None}] += ar
            if i == stride:  # generate precordial lead sequence
                ar = lead_group(sts=st_precordial, dt=dt, level=level)
                acanvas.x[{onset: None}] += ar
            onset += 60 / bpm
    return acanvas
```

Render and plot for a given or custom test condition

[6]:
```
# select your case -> choose index 0..4 in the next line
case = [
    "IE",
    "anterior_moderate",
    "anterior_severe",
    "inferior_moderate",
    "inferior_severe",
][3]
son_asig = render_ST_elevation_sonification_example(
    st_cabrera=sts_cabrera_dict[case],
    st_precordial=sts_precordial_dict[case],
)
plt.figure(figsize=(9, 3))
son_asig.plot(lw=0.25).stereo().play(onset=0.5)
plt.figure(figsize=(9, 3))
r = son_asig.to_stft().plot(pam.db_to_amp, cmap="hot", ylim=(0, 10000))

# uncomment to save the resulting sonification to a wave file
# son_asig.save_wavfile(f"ECG_STEMI_sonification_case={case}.wav")
```

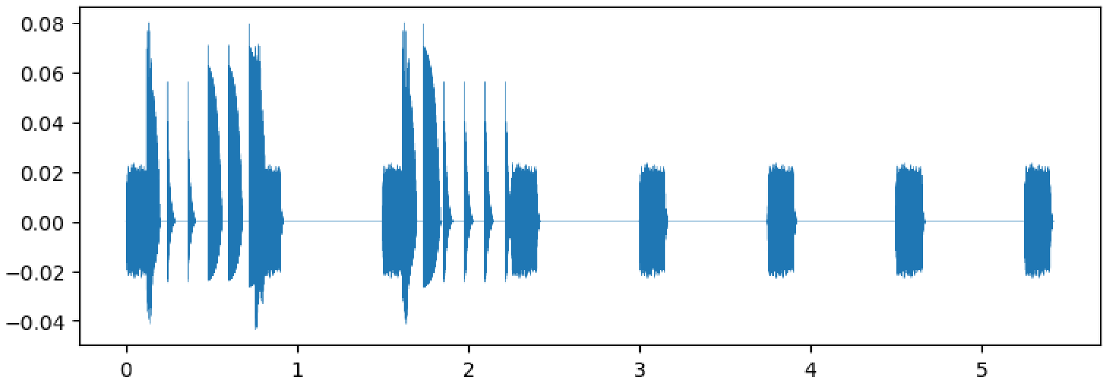

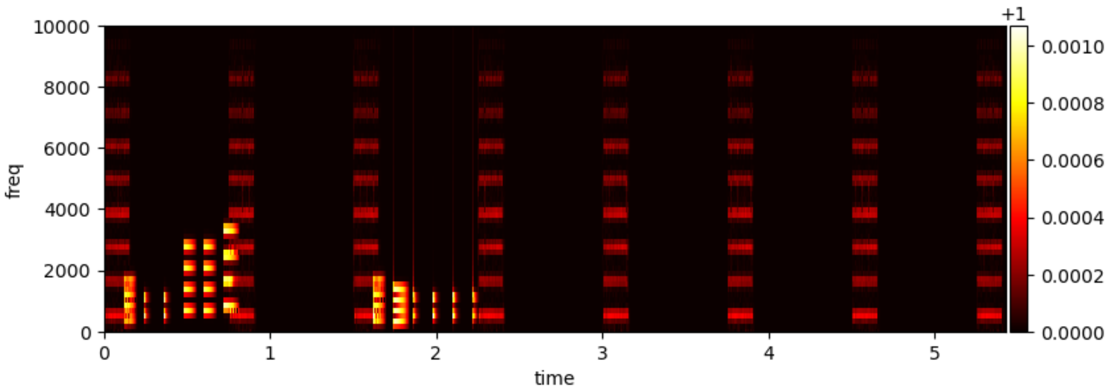

...or set a custom ST elevation profile

[7]:
```
son_asig = render_ST_elevation_sonification_example(
    st_cabrera=[-0.22, -0.12, -0.03, 0.03, 0.12, 0.24],
    st_precordial=[0.24, 0.13, 0.04, -0.05, -0.14, -0.24],
)
plt.figure(figsize=(9, 3))
son_asig.plot(lw=0.25).stereo().play(onset=0.5)
plt.figure(figsize=(9, 3))
r = son_asig.to_stft().plot(pam.db_to_amp, cmap="hot", ylim=(0, 10000))

# uncomment to save the resulting sonification to a wave file
# son_asig.save_wavfile(f"ECG_STEMI_sonification_case={case}.wav")
```

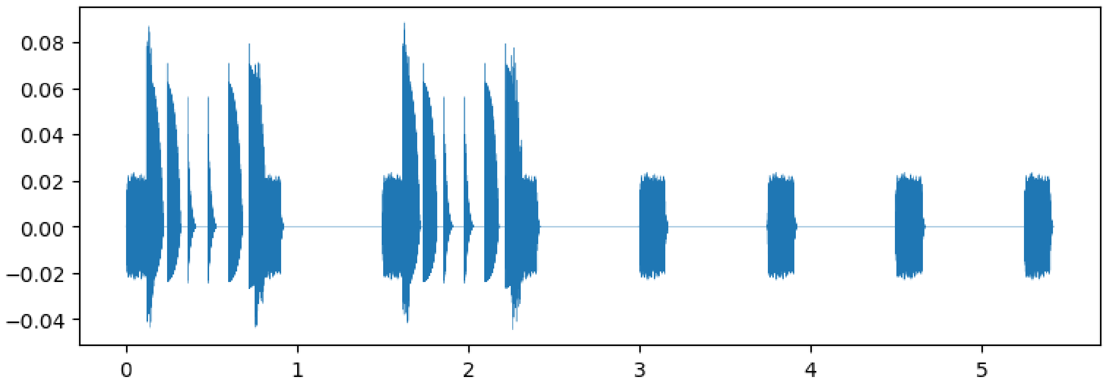
0.08
0.06
0.04
0.02
0.00
−0.02
−0.04
0
1
2
3
4
5

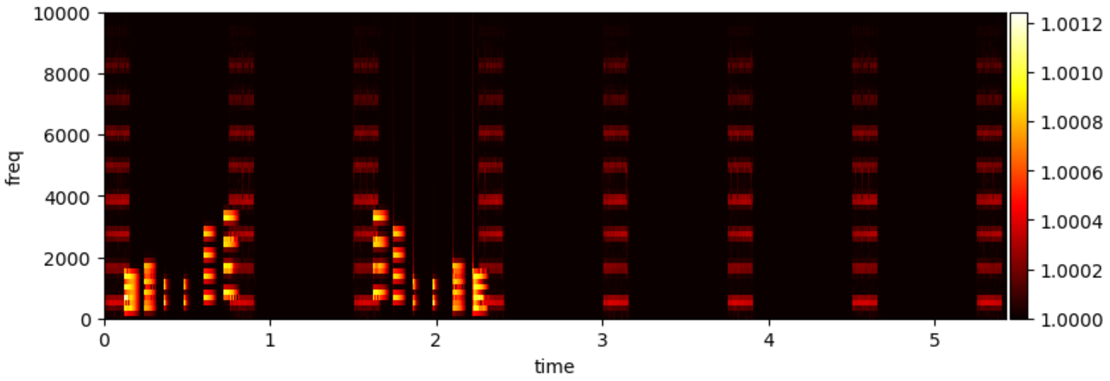
freq
time
10000
8000
6000
4000
2000
0
1
2
3
4
5
1.0012
1.0010
1.0008
1.0006
1.0004
1.0002
1.0000
